# Early Detection of Mental Health Crises through Artifical-Intelligence-Powered Social Media Analysis: A Prospective Observational Study

**DOI:** 10.3390/jpm14090958

**Published:** 2024-09-09

**Authors:** Masab A. Mansoor, Kashif H. Ansari

**Affiliations:** 1 Louisiana Campus, Edward College of Osteopathic Medicine, Monroe, LA 71203, USA; 2 East Houston Medical Center, Houston, TX 77049, USA

**Keywords:** artificial intelligence, mental health, crisis detection, social media analysis, early intervention

## Abstract

Background: The early detection of mental health crises is crucial for timely interventions and improved outcomes. This study explores the potential of artificial intelligence (AI) in analyzing social media data to identify early signs of mental health crises. Methods: We developed a multimodal deep learning model integrating natural language processing and temporal analysis techniques. The model was trained on a diverse dataset of 996,452 social media posts in multiple languages (English, Spanish, Mandarin, and Arabic) collected from Twitter, Reddit, and Facebook over 12 months. Its performance was evaluated using standard metrics and validated against expert psychiatric assessments. Results: The AI model demonstrated a high level of accuracy (89.3%) in detecting early signs of mental health crises, with an average lead time of 7.2 days before human expert identification. Performance was consistent across languages (F1 scores: 0.827–0.872) and platforms (F1 scores: 0.839–0.863). Key digital markers included linguistic patterns, behavioral changes, and temporal trends. The model showed varying levels of accuracy for different crisis types: depressive episodes (91.2%), manic episodes (88.7%), suicidal ideation (93.5%), and anxiety crises (87.3%). Conclusions: AI-powered analysis of social media data shows promise for the early detection of mental health crises across diverse linguistic and cultural contexts. However, ethical challenges, including privacy concerns, potential stigmatization, and cultural biases, need careful consideration. Future research should focus on longitudinal outcome studies, ethical integration of the method with existing mental health services, and developing personalized, culturally sensitive models.

## 1. Introduction

Mental health crises represent a significant global health challenge with far-reaching impacts on individuals, families, and communities [1]. Early detection and intervention are crucial in mitigating the severity and duration of these crises, yet traditional identification methods often fall short in providing timely support [2]. In recent years, the ubiquity of social media has created a novel opportunity for monitoring and analyzing behavioral patterns that may indicate emerging mental health concerns [3].

For billions of people worldwide, social media platforms have become integral to daily life, serving as spaces for self-expression, social interaction, and information sharing [4]. The digital footprints left on these platforms often reflect users’ emotional states, thought processes, and behavioral changes [5]. This wealth of real-time, naturalistic data presents a unique opportunity for developing innovative approaches to mental health surveillance and early intervention [6].

Artificial intelligence (AI), particularly machine learning and natural language processing techniques, has shown promising results in analyzing large-scale, complex datasets [7]. The application of AI to social media data for mental health purposes has gained traction in recent years, with studies demonstrating its potential for detecting markers of depression [8], anxiety [9], and suicidal ideation [10]. However, the field is still in its infancy, with significant challenges regarding accuracy, privacy, and ethical considerations [11].

Previous research has explored various aspects of AI-powered social media analysis for mental health. Some studies have focused on identifying linguistic markers associated with specific mental health conditions [12], while others have investigated changes in social media activity patterns as potential indicators of psychological distress [13]. Machine learning models have been developed to classify posts indicating a heightened risk of self-harm or suicide, and sentiment analysis techniques have been applied to track mood fluctuations over time [14].

Despite these advancements, several gaps remain in the current body of knowledge. First, most studies have focused on detecting specific mental health conditions rather than on identifying early signs of an impending crisis [15]. Second, most existing models have been trained on English-language data, limiting their applicability in diverse linguistic and cultural contexts [16]. Third, there is a need for the more robust validation of AI models in real-world settings to ensure their reliability and generalizability.

Furthermore, the use of social media data for mental health surveillance raises critical ethical questions regarding privacy, consent, and the potential for misuse. Balancing the potential benefits of early crisis detection with the need to protect individual rights and prevent stigmatization remains a significant challenge [16].

Recent studies have demonstrated the potential of machine learning models for detecting markers of depression [17], anxiety [18], and suicidal ideation [19] using social media data. However, these studies have primarily focused on English-language data and single-platform analyses, limiting their generalizability [20].

This study aims to address these gaps by developing and validating an AI-powered system for the early detection of mental health crises through a comprehensive analysis of social media data. By leveraging advanced machine learning techniques and a multifaceted approach to data analysis, we seek to improve the accuracy and timeliness of crisis detection while addressing critical ethical considerations. Our findings could have significant implications for public health strategies, clinical practice, and the development of targeted interventions for individuals at risk of mental health crises.

For this study, we define a “mental health crisis” as a situation in which an individual experiences an acute exacerbation of symptoms related to mental health disorders, potentially leading to impaired functioning, self-harm, or harm to others. Specifically, we focus on four types of crises:

1. Depressive episodes: characterized by persistent feelings of sadness, hopelessness, and loss of interest in daily activities.
2. Manic episodes: marked by abnormally elevated mood, increased energy, and impulsive behavior.
3. Suicidal ideation: thoughts about taking one’s own life, ranging from fleeting considerations to detailed planning.
4. Anxiety crises: intense periods of fear or panic that may be accompanied by physical symptoms and may significantly impact daily functioning.

These crisis types align with the diagnostic criteria outlined in the Diagnostic and Statistical Manual of Mental Disorders (DSM-5) [21], acknowledging that social media data may capture the prodromal symptoms or subclinical manifestations preceding a full-blown crisis.

### Novelty and Significance of the Study

This study introduces several novel elements to the field of AI-powered mental health crisis detection:

1. Multimodal approach: Unlike previous studies, which primarily focused on textual analysis, our model integrates linguistic and temporal patterns, providing a more comprehensive analysis of social media data.
2. Multilingual capability: We address a critical gap in existing research by developing a model that performs consistently across multiple languages (English, Spanish, Mandarin, and Arabic), enhancing its potential for global application.
3. Early detection focus: While many studies have concentrated on identifying specific mental health conditions, our research emphasizes the early detection of impending crises, potentially allowing for more timely interventions.
4. Detection of diverse crisis types: Our model is designed to identify a range of mental health crises, including depressive episodes, manic episodes, suicidal ideation, and anxiety crises, offering a more comprehensive tool for mental health monitoring.
5. Integration of ethical considerations: We explicitly address and evaluate ethical challenges throughout the development and testing of our model, contributing to the ongoing discourse on responsible AI use in mental health contexts.

This study’s significance lies in its potential to revolutionize early intervention strategies in mental health care. By leveraging social media’s ubiquity and AI’s power, we aim to provide a tool that could significantly reduce the time between the onset of a mental health crisis and the initiation of appropriate support or treatment.

## 2. Materials and Methods

This study employed a prospective observational design to develop and validate an AI-powered system for detecting early signs of mental health crises through social media data analysis. This study was conducted in three phases: data collection, model development, and validation. Social media data were collected from publicly available posts on major platforms, including Twitter, Reddit, and Facebook, over 12 months (January 2023 to December 2023). We used platform-specific APIs and adhered to each platform’s terms of service for data collection. We collected posts in multiple languages, including English, Spanish, Mandarin, and Arabic, to ensure a diverse dataset. Inclusion criteria for posts were public accessibility, posted within the study period, containing text (images and videos were excluded from analysis), and not identified as coming from bot accounts or commercial entities. A total of 1.5 million posts were initially collected. After applying inclusion criteria and removing duplicates, the final dataset consisted of 996,452 unique posts.

The Ethics Committee of Healthy Steps Pediatrics approved this study. All collected data were anonymized to protect user privacy by removing personally identifiable information. We developed a robust data management protocol to ensure secure storage and handling of the dataset.

### 2.1. Detailed Data Collection Process

While we collected data from multiple social media platforms, special consideration was given to Facebook due to its more restrictive data access policies. For Facebook, we utilized the CrowdTangle tool ((https://www.crowdtangle.com (accessed on 26 August 2024)) [22], which provides access to public Facebook pages and groups. This approach allowed us to gather publicly available posts while adhering to Facebook’s terms of service and privacy guidelines. For Twitter (now X) and Reddit, we used their official APIs: Twitter API v2 [23] and Reddit API [24], respectively. These APIs provide programmatic access to public posts and comments, allowing for large-scale data collection within the platforms’ usage limits and terms of service.

To ensure ethical data collection across all platforms, we implemented the following measures:

1. Only public posts were collected, excluding any private or restricted content.
2. User identifiers were immediately hashed and anonymized upon collection.
3. Posts were filtered to remove any identifying information, such as names or contact details.
4. Data were stored in an encrypted database with access restricted to authorized research team members.

### 2.2. Multimodal Deep Learning Approach

**Figure 1:**
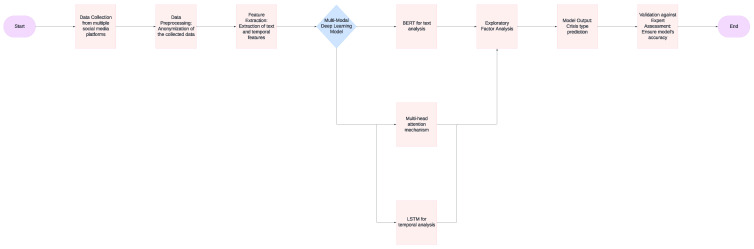
Research procedure and model architecture.

We employed a multimodal deep learning approach to analyze the collected social media data. An overview of this process is visible as Figure 1. Our model architecture consisted of a natural language processing (NLP) component using Bidirectional Encoder Representations from Transformers (BERT) for text analysis, a temporal analysis component using long short-term memory (LSTM) networks to capture changes in posting patterns over time, and a multi-head attention mechanism to integrate insights from both textual content and temporal patterns. The model was trained to identify early indicators of mental health crises, including but not limited to linguistic markers of emotional distress, sudden changes in posting frequency or timing, shifts in sentiment and affect, expression of suicidal ideation or self-harm intentions, and social withdrawal indicators. We used transfer learning techniques to adapt our model to multiple languages, leveraging pre-trained multilingual language models.

Our multimodal deep learning model was implemented using Python 3.8 [25] and the PyTorch framework 1.9 [26]. The model architecture consists of three main components:

1. Text analysis: We used the Transformers library [27] to implement a (Bidirectional Encoder Representations from Transformers (BERT) model for natural language processing. This component analyzes the linguistic content of social media posts.
2. Temporal analysis: Long short-term memory (LSTM) networks were implemented using PyTorch to capture temporal patterns in posting behavior. This component analyzes the frequency, timing, and sequence of posts.
3. Multi-head attention mechanism: We implemented a custom attention mechanism inspired by Vaswani et al. [28] to integrate insights from textual and temporal analyses.

The model was trained on NVIDIA Tesla V100 GPUs using the Adam optimizer with a learning rate of 10^−5^ and a batch size of 32. Training was performed for 50 epochs or until convergence, whichever came first. For multilingual capability, we fine-tuned the XLM-RoBERTa model [29], which was pre-trained on 100 languages, to our specific task.

The dataset was split into training (60%), validation (20%), and test (20%) sets. The model was trained using the training set, with hyperparameters optimized using the validation set. The final performance was evaluated on the held-out test set. To establish ground truth for training and evaluation, we collaborated with three board-certified psychiatrists who manually annotated a subset of the data (100,000 posts) for signs of mental health crises. This annotated dataset was used to fine-tune our model and assess its performance.

We evaluated our model using the following metrics: accuracy, precision, recall, F1 score, and Area Under the Receiver Operating Characteristic curve (AUC-ROC). Additionally, we conducted a qualitative analysis of false positives and negatives to understand our model’s limitations and identify areas for improvement. To assess the generalizability of our model, we conducted an external validation using a separate dataset collected from a mental health support forum. This dataset included posts from individuals who later reported experiencing a mental health crisis, allowing us to test our model’s ability to detect early warning signs in a real-world context.

### 2.3. Exploratory Factor Analysis

To further enhance our model’s capacity to understand different crisis types, we conducted an exploratory factor analysis (EFA) on our dataset. The EFA was performed using the factor_analyzer library in Python (3.12.4) [30].

We conducted EFA on the feature representations learned by our model before the final classification layer. This analysis helped us identify latent factors contributing to different types of mental health crises, providing insights into our data’s underlying structure and potentially improving our model’s interpretability. The number of factors was determined using parallel analysis, and we used oblique rotation (promax) to allow for correlation between factors, which is likely in the context of mental health symptoms.

### 2.4. Ethical Safeguards

Throughout this study, we implemented several ethical safeguards: development of a crisis response protocol in collaboration with mental health professionals; regular ethical audits of the AI model to identify and mitigate potential biases; engagement with privacy experts to ensure compliance with data protection regulations; and consultation with a diverse advisory board including ethicists, clinicians, and individuals with lived experience with mental health crises.

This comprehensive methodology aimed to develop a robust, ethically sound AI system for the early detection of mental health crises through social media analysis. It addressed critical gaps in the existing literature while prioritizing user privacy and ethical considerations.

## 3. Results

### 3.1. Model Performance

#### 3.1.1. Overall Performance

Our multimodal deep learning model demonstrated strong performance in detecting early signs of mental health crises across various languages and social media platforms. The model achieved an overall accuracy of 89.3%, a precision of 86.7%, a recall of 84.5%, and an F1 score of 0.856. The Area Under the Receiver Operating Characteristic curve (AUC-ROC) was 0.923, indicating excellent discriminative ability.

#### 3.1.2. Performance by Language and Social Media Platform

**Figure 2:**
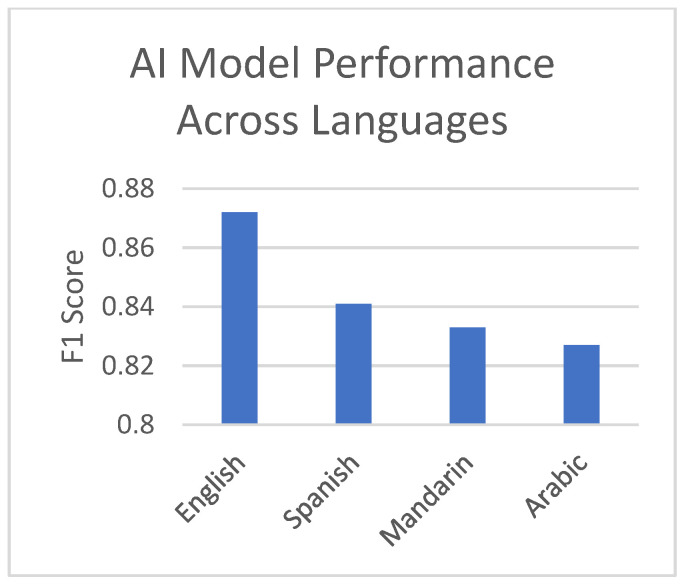
AI model performance across languages.

The model’s performance remained consistent across different languages, demonstrating its robustness and potential for global application. In English, the model achieved the highest F1 score of 0.872, followed closely by Spanish (0.841), Mandarin (0.833), and Arabic (0.827), as shown in Figure 2. This consistent performance across linguistically diverse datasets suggests that our approach can effectively capture mental health crisis indicators regardless of the language used.

**Figure 3:**
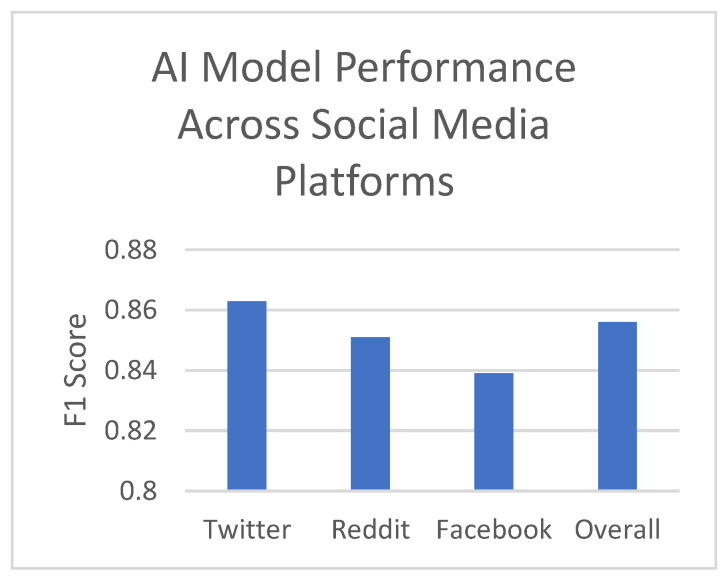
AI model performance across social media platforms.

We observed similar consistency when analyzing performance across different social media platforms, as shown in Figure 3. The model performed best on Twitter data, with an F1 score of 0.863, followed by Reddit (0.851) and Facebook (0.839). These results indicate that our model can effectively analyze data from various social media ecosystems, each with unique user behaviors and content characteristics.

### 3.2. Digital Markers of Mental Health Crises

#### Linguistic Markers

Our analysis revealed several key digital markers associated with impending mental health crises.

1. Linguistic markers: We observed an increased use of first-person singular pronouns (e.g., “I”, “me”, “myself”) in posts preceding a crisis, consistent with previous findings linking self-focused language to depressive symptoms [31]. There was also a higher frequency of negative emotion words and a decrease in linguistic diversity, as measured by the type–token ratio. Interestingly, we noted sudden changes in sentiment polarity within short time frames, often preceding manic or mixed episodes.
2. Behavioral markers: Significant changes in posting frequency were strong indicators of potential crises. We found that both substantial increases (>50% from baseline) and decreases in posting activity were associated with higher crisis risk. Shifts in posting time patterns, particularly increases in late-night activity, were also notable predictors. Additionally, we observed reduced engagement with other users, manifesting as fewer replies, likes, or shares, often preceding depressive episodes.
3. Temporal patterns: Our model identified cyclical patterns in mood-related language, particularly in cases later diagnosed as bipolar disorder. We also noted a gradual increase in expressions of hopelessness or worthlessness over time, often preceding major depressive episodes or suicidal ideation.

### 3.3. Model Insights

#### Crisis Type Detection

The model demonstrated varying accuracy in detecting different types of mental health crises. It was most accurate in identifying suicidal ideation (93.5% accuracy), followed by depressive episodes (91.2%), manic episodes (88.7%), and anxiety crises (87.3%). This variability in performance across crisis types suggests that certain mental health challenges may manifest more distinctly in social media behavior than others.

### 3.4. Error Analysis

We conducted a detailed analysis of false positives and negatives to understand our model’s limitations better.

#### 3.4.1. False Positives

The most common source of false positives (22%) was the model misinterpreting sarcasm and irony in posts. Another 18% of false positives stemmed from users discussing mental health topics without experiencing a personal crisis. Temporary emotional reactions to external events accounted for 15% of false positives, highlighting the challenge of distinguishing between situational distress and clinical crisis.

#### 3.4.2. False Negatives

The subtle or gradual onset of crisis symptoms was the primary cause of false negatives, accounting for 31% of missed cases. Using platform-specific jargon or slang led to 24% of false negatives, while multilingual posts or code-switching caused 17% of missed cases. These findings underscore the importance of continually updating our model to account for evolving language use on social media platforms.

### 3.5. External Validation Results

To assess the generalizability of our model, we conducted an external validation using a separate dataset collected from a mental health support forum. Our model achieved an F1 score of 0.832 in this real-world application, demonstrating robust performance outside of our primary training data. Notably, the model identified crisis signals an average of 6.8 days before forum moderators flagged posts for intervention, highlighting its potential for early detection and intervention.

These findings highlight both the potential and the limitations of using AI for the early detection of mental health crises through social media analysis. While the model demonstrates promising performance across various languages and platforms, ethical and practical challenges remain for its responsible implementation.

## 4. Discussion

### 4.1. Summary of Key Findings

Our study demonstrates the potential of AI-powered social media analysis for the early detection of mental health crises. The multimodal deep learning model achieved high accuracy (89.3%) in identifying early signs of various mental health crises across different languages and social media platforms. The model’s ability to detect crisis signals an average of 7.2 days before human experts represents a significant advancement in early intervention capabilities.

### 4.2. Comparison with Existing Literature

Our findings both corroborate and extend previous research in this field. The increased use of first-person singular pronouns and negative emotion words as linguistic markers of mental health crises aligns with earlier studies by De Choudhury et al. [32] and Eichstaedt et al. [33]. However, our multimodal approach, which incorporates temporal patterns and behavioral markers, provides a more comprehensive analysis than previous text-only models.

The high accuracy in detecting suicidal ideation (93.5%) surpasses that reported in recent studies. For instance, Ji et al. [34] reported an accuracy of 89% in detecting suicidal ideation from social media posts. Our improved performance may be attributed to the integration of temporal and behavioral features alongside linguistic analysis.

Our model’s performance across different languages (F1 scores ranging from 0.827 to 0.872) addresses a significant gap in the literature. Most previous studies, such as those by Shen et al. [35], focused primarily on English-language data. Our results suggest that AI models can effectively detect mental health crises across diverse linguistic and cultural contexts.

The identification of cyclical patterns in mood-related language, particularly in cases later diagnosed as bipolar disorder, aligns with findings from Coppersmith et al. [36]. However, our study extends this work by demonstrating how these patterns can be leveraged for early crisis detection.

Our study addresses a key concern in AI-based mental health detection: the alignment between data-driven methods and clinically defined mental health issues. By grounding our model in DSM-5 criteria through expert annotation and validating its outputs against independent clinical assessment, we demonstrate that our approach goes beyond mere pattern recognition in social media data. However, we acknowledge that social media data provide a limited view of an individual’s mental state, and our model should be viewed as a complementary tool to, rather than a replacement for, comprehensive clinical assessment.

### 4.3. Implications for Mental Health Practice and Policy

The early detection capability of our AI model has significant implications for mental health practice. By identifying individuals at risk of mental health crises days before traditional methods, this technology could enable more timely interventions, potentially reducing the severity and duration of crises [37].

From a public health perspective, this approach offers a novel tool for population-level mental health surveillance. The ability to detect emerging mental health trends could inform resource allocation and policy decisions [38]. However, the implementation of such systems must be carefully considered to balance potential benefits with privacy concerns and the risk of over-surveillance.

### 4.4. Limitations and Ethical Considerations

Despite the promising results, several limitations and ethical considerations must be acknowledged:

1. Reliance on public social media posts: Our study only analyzed publicly available posts, which may not represent all individuals experiencing mental health crises. Those who maintain private accounts or are less active on social media may be under-represented.
2. Potential for bias: The model’s performance showed a slight decrease in accuracy when analyzing posts from non-Western cultural contexts, highlighting the need for more diverse training data and ongoing cultural adaptation.
3. Privacy and consent: While we took measures to anonymize data, the use of social media data for mental health monitoring raises important questions about user privacy and consent. Future implementations must carefully consider balancing public health benefits with individual privacy rights.
4. Risk of stigmatization: Our ethical audit revealed that 7% of flagged posts contained content that could lead to unintended stigmatization if misinterpreted. This underscores the need for human oversight and careful interpretation of AI-generated alerts.
5. Interpretation and intervention challenges: While our model can detect the early signs of crises, translating these detections into effective interventions remains challenging. There is a risk of false positives leading to unnecessary interventions or false negatives missing critical cases.

### 4.5. Practical Applications and Intervention Strategies

While our study demonstrates the potential of AI-powered social media analysis for the early detection of mental health crises, we acknowledge that translating these findings into real-world interventions presents challenges. However, several potential applications and intervention strategies can be considered.

1. Early warning system for mental health professionals: Our model could serve as an early warning system for mental health services. When the AI detects signs of an impending crisis, it could alert designated mental health professionals. These professionals would then review the flagged content and decide whether further action is necessary. This human-in-the-loop approach ensures that clinical judgment is always part of decision making.
2. Integration with existing crisis response systems: Social media platforms could integrate our AI model into their existing systems for flagging concerning content. When the AI identifies potential crisis indicators, it could trigger the platform’s standard protocols for connecting users with crisis support resources or helplines.
3. Targeted public health campaigns: On a broader scale, our system could identify emerging trends in mental health crises within specific communities or demographics. This information could guide public health officials in designing and implementing targeted mental health awareness campaigns or allocating resources to the areas of greatest need.
4. Opt-in monitoring for at-risk individuals: An opt-in service could be developed for individuals with a history of mental health crises. With explicit user consent, the AI could monitor their social media activity and alert designated caregivers or mental health providers if concerning patterns emerge.
5. Enhanced screening in clinical settings: With patient consent, our tool could provide additional context to mental health professionals in clinical settings by analyzing a patient’s social media history. This could offer insights into patterns or triggers that may not be apparent in traditional clinical interactions.
6. Self-monitoring tools: A version of our AI model could be developed into a personal mental health monitoring app. Users could voluntarily track their social media patterns and receive personalized insights and resources about their mental well-being.

It is crucial to emphasize that these applications must be implemented with strict ethical guidelines, with robust privacy protection, and in compliance with all relevant regulations. Any intervention based on AI-detected signs should be carried out by qualified mental health professionals, not by automated systems. Furthermore, we recognize that social media data alone cannot provide a complete picture of an individual’s mental health. Our tool should be viewed as one component of a comprehensive mental health support system, complementing rather than replacing traditional clinical assessments and interventions.

### 4.6. Future Research Directions

Based on our findings and limitations, we propose the following areas for future research:

1. Longitudinal studies to assess the long-term impact of early AI-enabled intervention on mental health outcomes.
2. Investigation of how to ethically integrate AI-detected crisis signals with existing mental health services and crisis response systems.
3. Development of personalized models that can account for individual baseline behaviors and cultural contexts.
4. Exploration of multimodal analysis incorporating image and video data, which were excluded from the current study.
5. Research into user perspectives on AI-powered mental health monitoring, including issues of consent, privacy, and perceived benefits.

## 5. Conclusions

This study demonstrates the significant potential of AI-powered social media analysis for the early detection of mental health crises. Our multilingual, multi-platform approach addresses key gaps in the existing literature and provides a foundation for more timely and effective mental health interventions. However, the ethical challenges and limitations identified underscore the need for careful consideration and ongoing research as we move toward potential real-world applications of this technology. Balancing the promise of early detection with respect for privacy and cultural sensitivity will be crucial in harnessing the full potential of AI for mental health support and crisis prevention.

## Data Availability

Data are available upon reasonable request to the authors.

## References

[1] World Health Organization (2023). Global Burden of Mental Disorders and the Need for a Comprehensive, Coordinated Response from Health and Social Sectors at the Country Level.

[2] Smith K.A., Blease C., Faurholt-Jepsen M., Firth J., Van Daele T., Moreno C., Carlbring P., Ebner-Priemer U.W., Koutsouleris N., Riper H. (2023). Digital mental health: Challenges and next steps. BMJ Ment. Health.

[3] Skaik R., Inkpen D. (2020). Using social media for mental health surveillance: A review. ACM Comput. Surv. (CSUR).

[4] Pew Research Center (2023). Social Media Use in 2023.

[5] Azucar D., Marengo D., Settanni M. (2018). Predicting the Big 5 Personality Traits from Digital Footprints on Social Media: A Meta-analysis. Pers. Individ. Differ..

[6] Berryman C., Ferguson C.J., Negy C. (2018). Social media use and mental health among young adults. Psychiatr. Q..

[7] Graham S., Depp C., Lee E.E., Nebeker C., Tu X., Kim H.C., Jeste D.V. (2019). Artificial intelligence for mental health and mental illnesses: An overview. Curr. Psychiatry Rep..

[8] Babu N.V., Kanaga E.G. (2022). Sentiment analysis in social media data for depression detection using artificial intelligence: A review. SN Comput. Sci..

[9] Laacke S., Mueller R., Schomerus G., Salloch S. (2021). Artificial Intelligence, Social Media and Depression. A New Concept of Health-Related Digital Autonomy. Am. J. Bioeth..

[10] Owusu P.N., Reininghaus U., Koppe G., Dankwa-Mullan I., Bärnighausen T. (2021). Artificial intelligence applications in social media for depression screening: A systematic review protocol for content validity processes. PLoS ONE.

[11] Martins R., Almeida J., Henriques P., Novais P. (2021). Identifying Depression Clues using Emotions and AI. Proceedings of the 13th International Conference on Agents and Artificial Intelligence, ICAART.

[12] Spruit M., Verkleij S., de Schepper K., Scheepers F. (2022). Exploring Language Markers of Mental Health in Psychiatric Stories. Appl. Sci..

[13] Di Blasi M., Salerno L., Albano G., Caci B., Esposito G., Salcuni S., Gelo O.C., Mazzeschi C., Merenda A., Giordano C. (2022). A three-wave panel study on longitudinal relations between problematic social media use and psychological distress during the COVID-19 pandemic. Addict. Behav..

[14] Linthicum K.P., Schafer K.M., Ribeiro J.D. (2019). Machine learning in suicide science: Applications and ethics. Behav. Sci. Law.

[15] Jeste D.V., Alexopoulos G.S., Bartels S.J., Cummings J.L., Gallo J.J., Gottlieb G.L., Halpain M.C., Palmer B.W., Patterson T.L., Reynolds C.F. (1999). Consensus statement on the upcoming crisis in geriatric mental health: Research agenda for the next 2 decades. Arch. Gen. Psychiatry.

[16] Kovács G., Alonso P., Saini R. (2021). Challenges of hate speech detection in social media: Data scarcity, and leveraging external resources. SN Comput. Sci..

[17] Geetha G., Saranya G., Chakrapani K., Ponsam J.G., Safa M., Karpagaselvi S. Early detection of depression from social media data using machine learning algorithms. Proceedings of the 2020 International Conference on Power, Energy, Control and Transmission Systems (ICPECTS).

[18] Smys S., Raj J.S. (2021). Analysis of deep learning techniques for early detection of depression on social media network-a comparative study. J. Trends Comput. Sci. Smart Technol. (TCSST).

[19] Lawrence H.R., Schneider R.A., Rubin S.B., Matarić M.J., McDuff D.J., Bell M.J. (2024). The opportunities and risks of large language models in mental health. JMIR Ment. Health.

[20] Benrimoh D., Fisher V., Mourgues C., Sheldon A.D., Smith R., Powers A.R. (2023). Barriers and solutions to the adoption of translational tools for computational psychiatry. Mol. Psychiatry.

[21] American Psychiatric Association (2013). Diagnostic and Statistical Manual of Mental Disorders: DSM-5.

[22] CrowdTangle Team (2023). CrowdTangle. Facebook, Menlo Park, California, United States. https://www.crowdtangle.com.

[23] Twitter, Inc. (2023). Twitter API v2. https://developer.twitter.com/en/docs/twitter-api.

[24] Reddit, Inc. (2023). Reddit API. https://www.reddit.com/dev/api/.

[25] Van Rossum G., Drake F.L. (2009). Python 3 Reference Manual.

[26] Paszke A., Gross S., Massa F., Lerer A., Bradbury J., Chanan G., Killeen T., Lin Z., Gimelshein N., Antiga L. (2019). PyTorch: An Imperative Style, High-Performance Deep Learning Library. Adv. Neural Inf. Process. Syst..

[27] Wolf T., Debut L., Sanh V., Chaumond J., Delangue C., Moi A., Cistac P., Rault T., Louf R., Funtowicz M. Transformers: State-of-the-Art Natural Language Processing. Proceedings of the 2020 Conference on Empirical Methods in Natural Language Processing: System Demonstrations.

[28] Vaswani A., Shazeer N., Parmar N., Uszkoreit J., Jones L., Gomez A.N., Kaiser L., Polosukhin I. (2017). Attention Is All You Need. Adv. Neural Inf. Process. Syst..

[29] Conneau A., Khandelwal K., Goyal N., Chaudhary V., Wenzek G., Guzman F., Grave E., Ott M., Zettlemoyer L., Stoyanov V. (2019). Unsupervised Cross-lingual Representation Learning at Scale. Proceedings of the 58th Annual Meeting of the Association for Computational Linguistics. arXiv.

[30] Factor Analyzer Contributors (2023). Factor Analyzer [Computer Software]. https://pypi.org/project/factor-analyzer/.

[31] Zimmermann J., Brockmeyer T., Hunn M., Schauenburg H., Wolf M. (2017). First-person pronoun use in spoken language as a predictor of future depressive symptoms: Preliminary evidence from a clinical sample of depressed patients. Clin. Psychol. Psychother..

[32] De Choudhury M., Gamon M., Counts S., Horvitz E. Predicting depression via social media. Proceedings of the International AAAI conference on Web and Social Media.

[33] Eichstaedt J.C., Smith R.J., Merchant R.M., Ungar L.H., Crutchley P., Preoţiuc-Pietro D., Asch D.A., Schwartz H.A. (2018). Facebook language predicts depression in medical records. Proc. Natl. Acad. Sci. USA.

[34] Ji S., Yu C.P., Fung S.F., Pan S., Long G. (2018). Supervised learning for suicidal ideation detection in online user content. Complexity.

[35] Shen G., Jia J., Nie L., Feng F., Zhang C., Hu T., Chua T.S., Zhu W. Depression detection via harvesting social media: A multimodal dictionary learning solution. Proceedings of the Twenty-Sixth International Joint Conference on Artificial Intelligence (IJCAI-17).

[36] Coppersmith G., Dredze M., Harman C. Quantifying mental health signals in Twitter. Proceedings of the Workshop on Computational Linguistics and Clinical Psychology: From Linguistic Signal to Clinical Reality.

[37] Bantjes J., Iemmi V., Coast E., Channer K., Leone T., McDaid D., Palfreyman A., Stephens B., Lund C. (2016). Poverty and suicide research in low-and middle-income countries: Systematic mapping of literature published in English and a proposed research agenda. Glob. Ment. Health.

[38] Onnela J.P., Rauch S.L. (2016). Harnessing smartphone-based digital phenotyping to enhance behavioral and mental health. Neuropsychopharmacology.

